# Exercise training modulates the gut microbiota profile and impairs inflammatory signaling pathways in obese children

**DOI:** 10.1038/s12276-020-0459-0

**Published:** 2020-07-06

**Authors:** Rocío Quiroga, Esther Nistal, Brisamar Estébanez, David Porras, María Juárez-Fernández, Susana Martínez-Flórez, María Victoria García-Mediavilla, José A. de Paz, Javier González-Gallego, Sonia Sánchez-Campos, María J. Cuevas

**Affiliations:** 1 Complejo Asistencial Universitario (CAULE), León, Spain; 2 Institute of Biomedicine (IBIOMED), León, Spain; 3 Centro de Investigación Biomédica en Red de Enfermedades Hepáticas y Digestivas (CIBERehd), Madrid, Spain

**Keywords:** High-throughput screening, DNA metabolism

## Abstract

Childhood obesity has reached epidemic levels and is a serious health concern associated with metabolic syndrome, nonalcoholic fatty liver disease, and gut microbiota alterations. Physical exercise is known to counteract obesity progression and modulate the gut microbiota composition. This study aims to determine the effect of a 12-week strength and endurance combined training program on gut microbiota and inflammation in obese pediatric patients. Thirty-nine obese children were assigned randomly to the control or training group. Anthropometric and biochemical parameters, muscular strength, and inflammatory signaling pathways in mononuclear cells were evaluated. Bacterial composition and functionality were determined by massive sequencing and metabolomic analysis. Exercise reduced plasma glucose levels and increased dynamic strength in the upper and lower extremities compared with the obese control group. Metagenomic analysis revealed a bacterial composition associated with obesity, showing changes at the phylum, class, and genus levels. Exercise counteracted this profile, significantly reducing the *Proteobacteria* phylum and *Gammaproteobacteria* class. Moreover, physical activity tended to increase some genera, such as *Blautia, Dialister*, and *Roseburia*, leading to a microbiota profile similar to that of healthy children. Metabolomic analysis revealed changes in short-chain fatty acids, branched-chain amino acids, and several sugars in response to exercise, in correlation with a specific microbiota profile. Finally, the training protocol significantly inhibited the activation of the obesity-associated NLRP3 signaling pathway. Our data suggest the existence of an obesity-related deleterious microbiota profile that is positively modified by physical activity intervention. Exercise training could be considered an efficient nonpharmacological therapy, reducing inflammatory signaling pathways induced by obesity in children via microbiota modulation.

## Childhood obesity: working out for a healthier microbiome

Physical fitness regimens could stimulate shifts in gut microbiome composition and metabolism that counteract health risks associated with childhood obesity. This condition can increase the risk of metabolic and cardiovascular disease later in life. Several studies have indicated that disturbances in the microbial populations in the digestive tract may contribute to these diseases. Researchers led by Sonia Sánchez-Campos of the Institute of Biomedicine, León,Spain, recently assessed the impact of exercise and endurance training on microbiome composition in obese children. They determined that these regimens can produce measurable shifts in the gut microbe population, yielding bacterial communities that are more similar to those seen in non-obese children. These shifts were accompanied by alterations in metabolic activity that may help mitigate inflammatory signaling and other processes that fuel obesity-related disease.

## Introduction

Obesity among children, adolescents, and adults are one of the most serious public health concerns since it has been associated with many other diseases, including cardiovascular and respiratory diseases and type 2 diabetes^1,2^. Until a few decades ago, obesity was a problem that developed in the mature stage of an individual’s life. However, obesity is currently affecting children and adolescents, generating these obesity-associated diseases in early stages and increasing the risk of obesity in adulthood^1,3^. Obesity is a complex, multifactorial condition affected by genetic and nongenetic factors^2^. Among them, intestinal microbiota has been shown to be a major factor underlying obesity in adults^4,5^, and recent research also links changes in microbiota composition to obesity in children^6,7^. Childhood is a critical stage in the development of the microbiota due to the great plasticity of the gut ecosystem in this period, influenced by environmental events that take place in early life, including infant feeding, antibiotic/probiotic exposure or early dietary patterns^8^.

The homeostasis of the intestinal microbiota depends on many factors related to the characteristics of the host, such as age, sex, and genetic background, as well as environmental conditions^9^. In the last few years, several researchers, including our group, have begun to study the influence that alteration of the intestinal microbiota, also called intestinal dysbiosis, can have on the development of obesity and related pathogenic alterations^10–12^. In fact, the intestinal microbiota has an important role in obesity, as the efficiency of the digestion of nutrients as well as storage and energy expenditure depend on the microbiota composition^13^.

Furthermore, it is also important to highlight that changes in the microbiota composition are known to provide potential harmful molecules that disrupt gut integrity, inducing a systemic inflammatory response mediated by pattern recognition receptors, thus contributing to the establishment of a chronic low-grade inflammation state associated with obesity^10,11,14^. Evidence demonstrates that there is a correlation between gut microbiota, energy homeostasis, and the development of inflammation in the pathogenesis of alterations related to obesity^15^.

In the case of obese children, the vast majority of studies suggest that physical exercise is the best tool to reduce abdominal obesity and cardiovascular risk and improve metabolic parameters^16^. Moreover, the modulation of intestinal microbiota by physical exercise improves the progression of diseases associated with dysbiosis, maintains gut barrier integrity, and counteracts the inflammatory response^17,18^, even in an in vivo model of early obesity^10,19^. Although there are divergences in the methodology used, it has been shown that the application of a combined training program of strength and endurance exercise is able to reduce body fat and waist circumference to a greater extent than the use of one of them individually^20^.

The aim of our study was to determine whether a combined 12-week endurance and strength training protocol could favorably modify intestinal microbiota composition and functionality and their inflammatory status in children with obesity.

## Materials and methods

### Participants and ethics approval

Fifty-three subjects aged between 7 and 12 years old were included in this study: thirty-nine obese pediatric patients (O) and fourteen healthy control subjects (Hc) without signs of pubertal development were selected and recruited from the Pediatric Endocrinology consultation (Complejo Asistencial Universitario de León, CAULE) to participate. The obese pediatric group was randomly split into two categories based on whether the participants underwent the training protocol or not. Training participants (Oe, *n* = 25) followed a 12-week combined strength and endurance training program, whereas the control obese group (Oc, *n* = 14) maintained their normal daily routines. Finally, five patients in each obese group left the study for different personal reasons (Supplementary Fig. 1).

Exclusion criteria included diseases and disabilities that limit the performance of physical exercise, medication that affects the intake of food or appetite, or antibiotic, prebiotic, probiotic, or symbiotic treatments that could affect the gut microbiota composition. Subjects who had genetic disorders, active autoimmune or chronic diseases, diabetes or acute diseases or children who had required bed rest in the last 3 months or were participating in after-school physical activity programs were also excluded. The study was conducted according to the guidelines outlined in the Declaration of Helsinki, and all procedures involving human subjects were approved by the local ethics committee of our hospital. The parents or guardians of each child signed the informed consent form for participation, and all children assented to participate.

### Experimental design and maximal strength assessment

This study was completed in 14 weeks. Subjects performed a combined strength and endurance exercise training program for 12 weeks, and baseline data were collected 1 week prior (Pre) and after (Post) the exercise protocol. All the participants underwent full-body densitometry performed by dual X-ray absorptiometry (Densitometer, GE Lunar Prodigy), anthropometric analysis, and assessment of a one-repetition maximum (1RM). In weeks 1 and 14, and after a standardized 10-min warm up on a cycle ergometer (Tunturi F35, Tunturi^®^, Turku, Finland), the 1RM of the leg press (45°-inclined leg press device; Gervasport, Madrid, Spain) and seated chest press (BH Fitness Nevada Pro, BH, Vitoria, Spain) was evaluated, following the methodology described by Faigenbaum et al.^21^.

### Combined strength and endurance training program

Subjects completed 24 combined strength and endurance exercise training sessions over 12 weeks (2 sessions per week), with at least 48 h between sessions. Each session began with a general warm-up performed on a cycle ergometer for 7 min with a low-medium load and a rate of 60 rpm. From the third minute, the participating children were asked to perform a sprint of 30 s at a maximum cadence at 3′30”, 4′30”, 5′30”, and 6′30”, progressively increasing the load level after each sprint. Next, each of the children performed the strength exercises (leg press, knee extension, pectoral press, and pectoral contractor). Strength work was done on four different strength machines, working five muscle groups. They started the program doing 3 sets of 12 repetitions with a load of 30% 1RM; progressively, this load was increased until performing 3 sets of 8 repetitions with a load of 70% 1RM. Between each series, a break of between 30 and 60 s was established. The protocol ended with the performance of elliptical cardiovascular exercise for 7 min at a rate of 50 rpm. The load was low-medium during the first 4 min, and the load was high in the last 3 min (Reebok GX60, Canton MA, USA). The total exercise time developed in each session was 45 min (Table 1).

**Table 1:** Training protocol.

Warm-up				
	Series	Intensity	Cadence	Volume
Cycloergometer	1	3/10 Max for 30” every min	60 rpm	7’

Strength training				
	Series	Repeat	Intensity	Break
Leg press	3	12–10	30–70% 1RM	1’
Knee extension	3	12–8	30–70% 1RM	30”
Pectoral press	3	12–8	30–70% 1RM	30”
Pectoral contractor	3	12–8	30–70% 1RM	30”

Endurance training				
	Series	Intensity	Cadence	Volume
Elliptical	11	6/8	50 rpm	4’
Elliptical		20/32	50 rpm	3’

*RM* maximum dynamic strength, *rpm* revolutions per minute.

### Gut microbiota analysis

Fresh stools from the different groups considered in the childhood obesity model were collected the week before and after the completion of the physical training protocol. All samples were homogenized and aliquoted within 3 h of defecation. The aliquots were stored at −80 °C until analysis.

Genomic DNA was extracted from 200 mg of the different fecal samples using the QIAamp DNA Stool Mini kit (Qiagen, Hilden, Germany), with some modifications. The lysis temperature was increased from 70 to 90 °C, and an initial bead-beating step was included to aid in the recovery of DNA from bacteria that are difficult to lyse. The concentration of DNA extracted from each of the samples was determined using a NanoDrop ND-1000 spectrophotometer (Saveen & Werner, Limhann, Sweden). Amplification of the 16 S rRNA V3-V4 hypervariable region was carried out using the 16 S V3 314 F forward (5′TCGTCGGCAGCGTCAGATGTGTATAAGAGACAGCCTACGGGNGGCWGCAG3′) and V4 805 R reverse primers (5′GTCTCGTGGGCTCGGAGATGTGTATAAGAGACAGGACTACHVGGGTATCTAATCC3′)^12^ with a specific adapter for subsequent sequencing with the *Illumina* MiSeq system. For each individual, three PCRs were carried out, and the resulting amplicons were mixed, cleaned, quantified, and sequenced on the Illumina MiSeq platform.

For bioinformatics analysis, samples were evaluated using BaseSpace Application 16 S Metagenomics v1.0 (Illumina Inc.). To confirm the results, “Quantitative Insights into Microbial Ecology” software (QIIME version 1.9.0) was utilized^12^. Processed reads were then clustered in operational taxonomic units (OTUs) using UCLUST with a similarity threshold of 0.97^22^. Subsequently, OTUs were aligned using PyNast^23^ against the 16 S reference database GreenGenes (version 13.8) using default parameters. These OTUs were interpreted with the Vegan package (https://CRAN.R-project.org/package=vegan) in R software (R Development Core Team, 2011) to assign the alpha and beta diversity of the samples.

### Metabolomic analysis

For the extraction procedure, ~70–100 mg of fecal dry matter content was used, and 1000 µl of MeOH:H_2_O (8:1) was added. The sample was vortexed (1 min), sonicated and then centrifuged (15 min at 4 °C, 20,000 × *g*), separating into an upper clear phase and a lower turbid phase (with lipids, proteins, and cellular debris). The clear upper phase (700 µl) was transferred into a new vial and dried under an N_2_ stream. The sample was resuspended in 600 µl of buffer containing 2.32 mM TSP and 0.05 M PBS (Na_2_HPO_4_ and NaH_2_PO_4_) in D_2_O and transferred to a 5-mm NMR tube.

The 1H NMR spectra were acquired at 300 K on an Advance III 500 spectrometer (Bruker®, Germany) operating at a proton frequency of 500.20 MHz, using a 5-mm TXI probe. One-dimensional 1H pulse experiments were carried out using the nuclear Overhauser effect spectroscopy (NOESY)-presaturation sequence to suppress the residual water peak at around 4.7 ppm. The spectral width was 20 ppm, and a total of 128 k data points were collected for each spectrum. Using TopSpin software (Bruker^®^), the spectra were manually phased and baseline-corrected before performing automatic metabolite profiling of the spectra through adaptation of Dolphin^24^. Several database engines (BBioref AMIX database (Bruker), Chenomx and HMDB^25^) were used for 1D-resonance assignment and metabolite identification.

Metabolomics data analysis was performed with the Metaboanalyst 4.0 platform^26^. After data pretreatment and standardization, partial least squares-discriminant analysis (PLS-DA) models were established.

### Venous blood sampling

Blood samples (5–10 ml) were obtained using the Vacutainer^®^ system (BD, Franklin Lakes, NJ), with EDTA as an anticoagulant from the brachiocephalic vein in the early morning in the fasted state at the Pediatrics and Neonatology office of CAULE 5–6 days before and after the training period. Peripheral blood mononuclear cells were isolated from whole blood by density gradient centrifugation on Ficoll separating solution (Biochrom AG, Berlin, Germany).

### Standardized determination of hematological and biochemical parameters

The determination of hematological and biochemical parameters was carried out in the Clinical Analysis service of CAULE. The hematological examination consisted of the counting of blood cells and leukocyte subpopulations. The biochemical parameters determined were the lactate dehydrogenase (LDH), gamma glutamyl transpeptidase, glutamate-oxaloacetate transaminase (GOT), and glutamate-pyruvate transaminase activities; total and direct bilirubin plasma concentration; glycemia; insulin; Homeostatic Model Assessment-Insulin Resistance index; and the high-density lipoprotein, low-density lipoprotein, triglycerides, and C reactive protein levels. Plasma lipopolysaccharide (LPS) levels were determined by the Limulus Amebocyte Lysate Chromogenic Endotoxin Quantitation Kit (Thermo Scientific, Waltham, US).

### Western blot analysis

PBMCs were homogenized in 150 μl of buffer containing 0.25 mM sucrose, 1 mM EDTA, 10 mM Tris, and a protease inhibitor cocktail (Sigma-Aldrich, St. Louis, MO, USA) with an ultrasonic processor (UP100H, Hielscher, Teltow, Germany). The protein content of each sample was measured by the technique described by Bradford. Samples containing 40 μg of protein were separated by SDS-PAGE on 10% SDS-polyacrylamide gels. Separated proteins were transferred to PVDF membranes, and nonspecific binding was blocked by preincubation of the membranes in 5% nonfat milk-PBS for 30 min at 37 °C. After that, incubation with specific primary antibodies was performed overnight at 4 °C^27^. Antibodies against caspase-1 (CASP-1) (50 kDa, Ref. 2225) and the NLR family pyrin domain containing 3 (NLRP3) (110 kDa, Ref. 15101) were purchased from Cell Signaling Technology^®^; osteopontin (OPN) (66 kDa, Ref. ab8448) was purchased from Abcam^®^, Cambridge, UK, USA; Toll-like receptor 4 (TLR4) (95 kDa, Ref. sc-293072) was purchased from Santa Cruz Biotechnology, CA, USA; and β-actin (42 kDa, Ref. A5060), which served as a control protein, was purchased from Sigma-Aldrich, St. Louis, MO, USA. Bound primary antibody was detected using a peroxidase-conjugated secondary antibody (Dako, Glostrup, Denmark) and an enhanced chemiluminescence-HRP kit (Luminol Reagent Santa Cruz Biotechnology). The density of the specific bands was quantified with an imaging densitometer (ImageJ, Bethesda, MD, USA).

### Statistical analysis

Significant differences in the gut microbiota composition were tested by the Kruskal–Wallis test followed by the Mann–Whitney U test when *p* < 0.05. Correlations between gut microbiota composition and fecal metabolites were examined by Spearman’s correlation using the microbiome package in R software (http://microbiome.github.io/microbiome/). Correlations with a *p* value < 0.05 were selected to construct the network map. The rest of the values are presented as the mean ± standard error of the mean. Post-training values were normalized to pretraining values. The Shapiro–Wilk test was used to verify normal data distribution. When the samples presented a normal distribution, data were analyzed using a two-way analysis of variance with repeated measures for group (trained vs untrained) and time (pre and post). Bonferroni analysis was used to compensate for multiple post hoc comparisons. Differences were considered significant when *p* < 0.05. All statistical analyses were performed using SPSS version 22.0 (SPSS Inc., Chicago, IL, USA).

## Results

### Anthropometric, hematological, and biochemical characteristics in obese children

The anthropometric, hematological, and most of the biochemical data showed no significant differences between the control and trained obese groups throughout the study (data not shown). Nevertheless, exercise training reduced glucose levels and GOT and LDH activities after a 12-week strength and endurance combined training program (Supplementary Fig. 2).

### Dynamic strength

Dynamic strength was significantly increased by physical exercise in the training group (Oe) compared with the control group (Oc) in the upper and lower extremities (Table 2). In the sedentary group, there were no significant differences in maximum dynamic strength (RM) between the beginning (*t* = 0 weeks) and the end of the study (*t* = 12 weeks). Nevertheless, physical exercise significantly increased the maximum dynamic strength of the upper and lower extremities after 12 weeks of exercise in the training group in contrast to the control group. Related to the % ∆RM, this parameter was increased two times and four times in the lower extremities and upper extremities, respectively, because of the exercise intervention in the training group compared with the control group (Table 2).

**Table 2:** Values of strength in obese control (Oc) and training group (Oe) before (*t* = 0) and after (*t* = 12 ws) exercise intervention on superior (EESS) and inferior (EEII) extremities. Maximum dynamic (RM) strength and percentage change after exercise intervention (% ∆RM).

	Control group (Oc)		Training group (Oe)	
	*t* = 0	*t* = 12 ws	*t* = 0	*t* = 12 ws
RM EESS (kg)	45.6 ± 2.7	50.7 ± 2.8	48.2 ± 3.5	71.8 ± 2.7^*** ##^
% ∆RM EESS	12.9 ± 3.3		61.4 ± 17.4^**^	
RM EEII (kg)	123.5 ± 7.5	138.3 ± 9.4	160.8 ± 9.5^**^	203.6 ± 11.3^*** ###^
% ∆RM EEII	15.3 ± 3.4		31.6 ± 4.4^*^	

Values are presented as mean ± SEM.

*p < 0.05 vs Oc (*t* = 0); **p < 0.01 vs Oc (*t* = 0); ***p < 0.001 vs Oc (*t* = 0); ^##^*p* < 0.01 vs Oe (*t* = 0); ^###^*p* < 0.001 vs Oe (*t* = 0)

### Gut microbiota composition

**Fig. 1:**
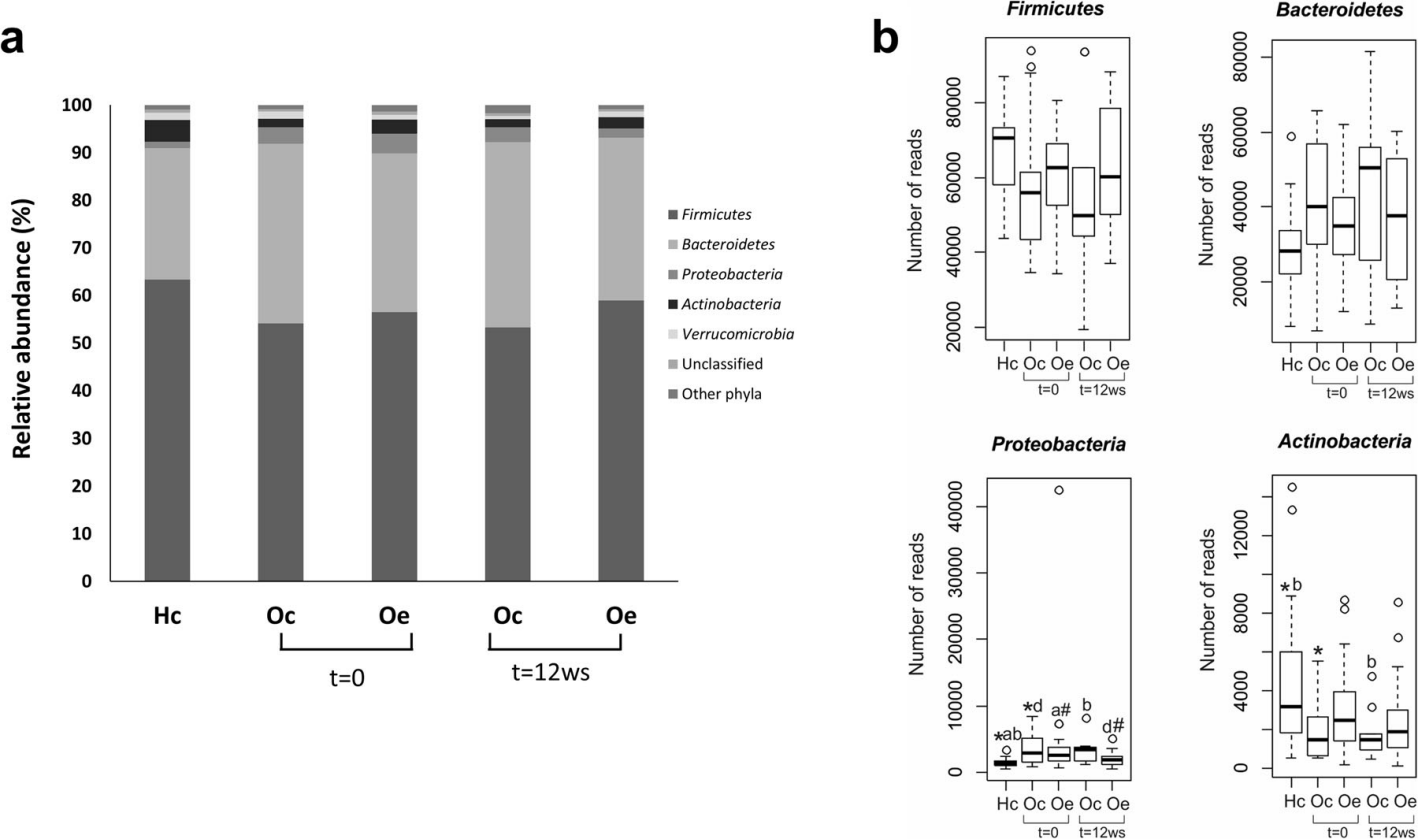
Comparison of the phylotypes between healthy control children (Hc) and obese patients assigned randomly to a control (Oc) or a training (Oe) group at the beginning (*t* = 0) and at the end of the study (*t* = 12 weeks) at the phylum level. **a** Bar graphs represent the relative abundance of the total population. **b** Box plot summarizing significant differences using the Kruskal–Wallis test followed by the Mann–Whitney U test (*p* < 0.05). ^*^Hc vs Oc (*t* = 0), ^a^Hc vs Oe (*t* = 0), ^b^Hc vs Oc (*t* = 12 weeks), ^d^Oc (*t* = 0) vs Oe (*t* = 12 weeks), ^#^Oe (*t* = 0) vs Oe (*t* = 12 weeks).

A total of 9,010,771 bacterial sequences were obtained from the analysis of 84 fecal samples, ranging from 58,320 to 198,123 reads per sample. Analysis of the sequences indicated that this niche was colonized by bacteria affiliated mainly with four phyla. The *Firmicutes* (58%) and *Bacteroidetes* (34%) phyla were the most representative, followed by *Proteobacteria* (2.6%) and *Actinobacteria* (2.8%). Figure 1a shows the relative bacterial composition at the phylum level for each group. Compared with healthy control children, we observed a bacterial profile associated with obesity, showing a higher detection of the *Bacteroidetes* and *Proteobacteria* phyla, while *Firmicutes* and *Actinobacteria* showed an opposite pattern (Fig. 1a). Moreover, statistical analysis revealed that these differences were significant in the number of reads of the *Proteobacteria* and *Actinobacteria* phyla compared with those of the healthy controls (Fig. 1b). On the other hand, exercise performance tended to counteract this microbiota profile associated with obesity, significantly reducing the *Proteobacteria* phylum, which tended to be similar to that found in healthy control children (Fig. 1a,b).

**Fig. 2:**
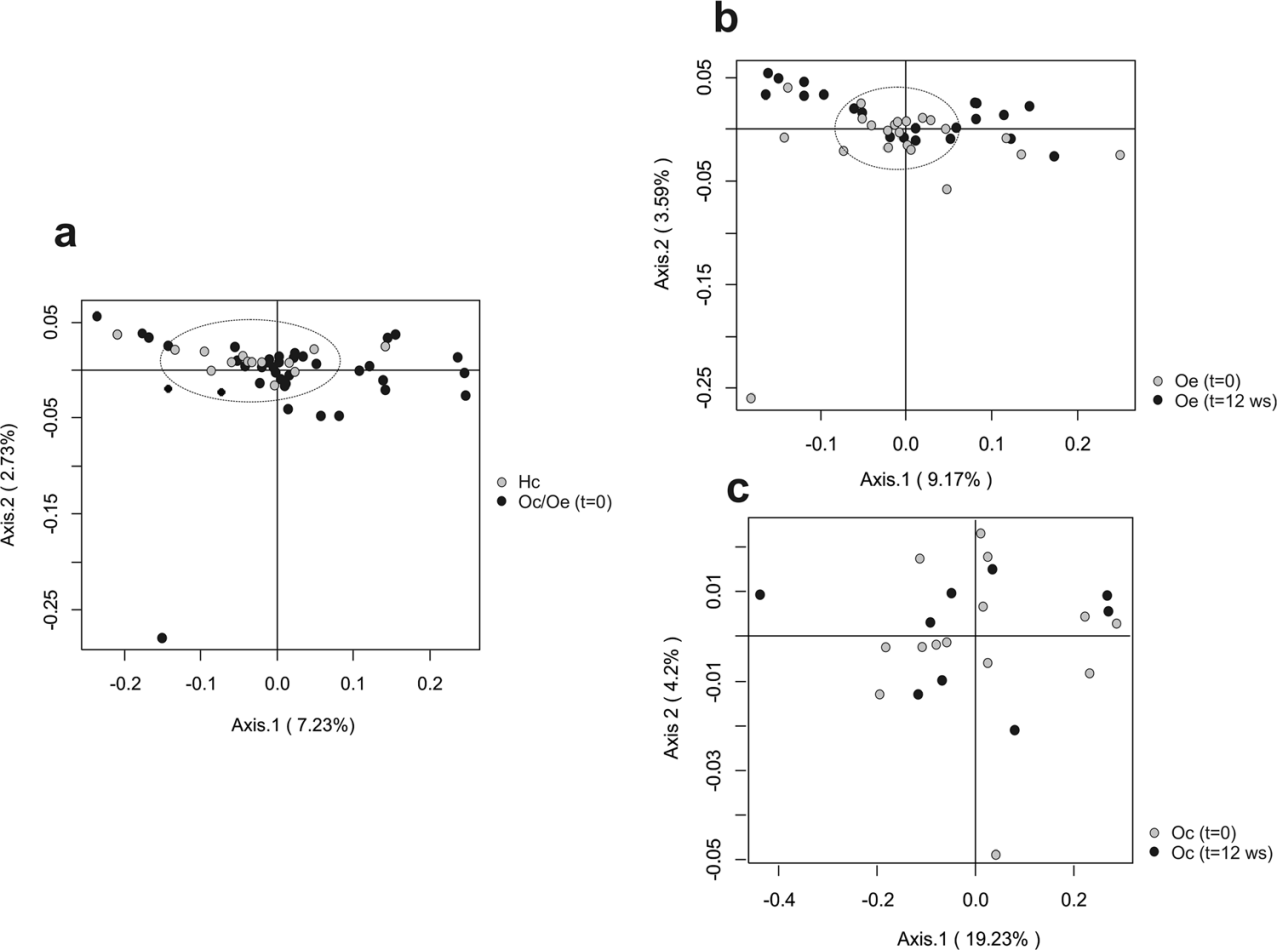
Principal coordinates analysis (PCoA) plot derived from the Morisita-Horn index at the phylum level. The percentage of the total variance explained is indicated in parentheses in each axis. Dashed lines denote sample clusters according to obesity or exercise. **a** Comparison of bacterial communities between healthy control children (Hc) and obese patients (Oc and Oe) at the beginning of the study (*t* = 0). **b** Effect of exercise on the bacterial communities comparing trained obese patients (Oe, *t* = 12 weeks) with the corresponding group at the beginning of the study (Oe, *t* = 0). **c** PCoA comparing bacterial communities longitudinally in obese control patients (Oc, *t* = 0, *t* = 12 weeks).

Principal coordinates analysis based on the Morosita-Horn Index was performed to analyze the influence of obesity and exercise factors on microbiota distribution at the phylum level. This analysis, considering only fecal samples at the beginning of the study, revealed that the bacterial communities of the healthy controls group separated from those of obese patients according to the first axis. This component accounted for 7.23% of the total variance (Fig. 2a). Conversely, comparing trained obese patients with their corresponding group at the beginning of the study, the first axis score plot (9.17%) denoted that exercise performance was found to be a dispersing factor (Fig. 2b). No grouping factor was observed when comparing bacterial communities longitudinally for obese control patients (Fig. 2c). In addition, comparing fecal microbiota from healthy control children and obese patients after the exercise period, the results revealed that the bacterial communities of trained obese patients clustered together with the bacterial communities of healthy control children (Supplementary Fig. 3a). However, comparing obese control and obese trained patients at the end of the study, exercise performance was found to be a dispersing factor, while the bacterial communities of obese control patients were grouped separately (Supplementary Fig. 3b).

**Fig. 3:**
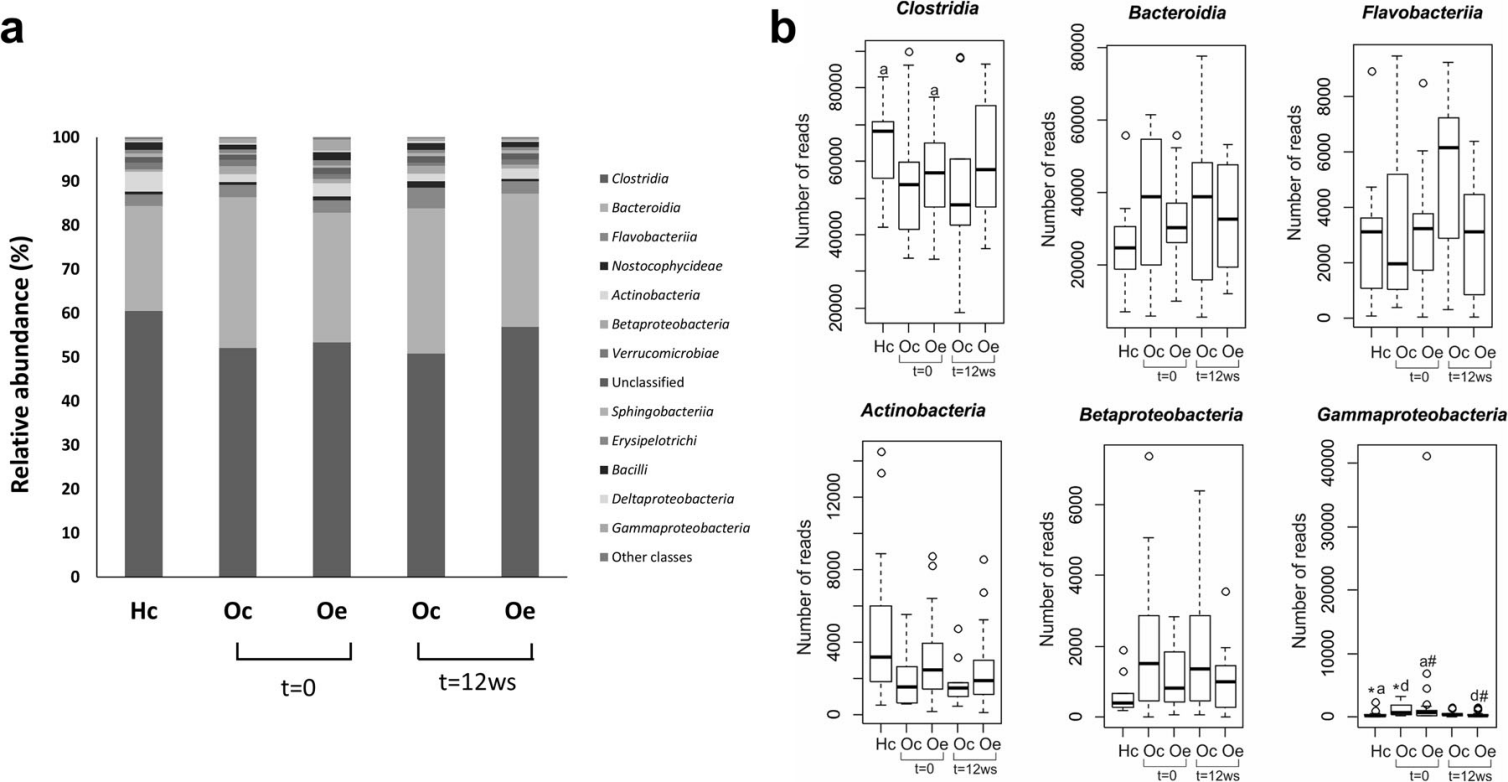
Gut microbiota composition at the class level. **a** Bar graphs representing the bacterial community composition in healthy controls (Hc) and obese patients randomly assigned to a control (Oc) or a training (Oe) group. **b** Box plot showing the significant differences at the class level using the Kruskal–Wallis test followed by the Mann–Whitney U test (*p* < 0.05). ^*^Hc vs Oc (*t* = 0), ^a^Hc vs Oe (*t* = 0), ^d^Oc (*t* = 0) vs Oe (*t* = 12 weeks), ^#^Oe (*t* = 0) vs Oe (*t* = 12 weeks).

At the class level, *Clostridia* and *Actinobacteria* (classified within the *Firmicutes* and *Actinobacteria* phyla, respectively) were considerably reduced in obese patients in comparison to the healthy control group, while *Bacteroidia* (*Bacteroidetes* phylum), *Gammaproteobacteria* and *Betaproteobacteria* (both classified within the *Proteobacteria* phylum) showed the opposite pattern (Fig. 3a). In addition, the relative abundance of these last two classes was significantly different between obese patients and healthy control children. Compared with obese control patients, exercise training for 12 weeks was found to substantially modify the relative abundance of *Clostridia*, *Flavobacteriia* (*Bacteroidetes* phylum), *Actinobacteria* and *Gammaproteobacteria*, revealing a microbiota profile at the class level that tended to be similar to the healthy control group (Fig. 3a). Significant differences associated with exercise performance were obtained for the *Gammaproteobacteria* class in obese trained patients compared with the corresponding group at the beginning of the study (Fig. 3b).

**Fig. 4:**
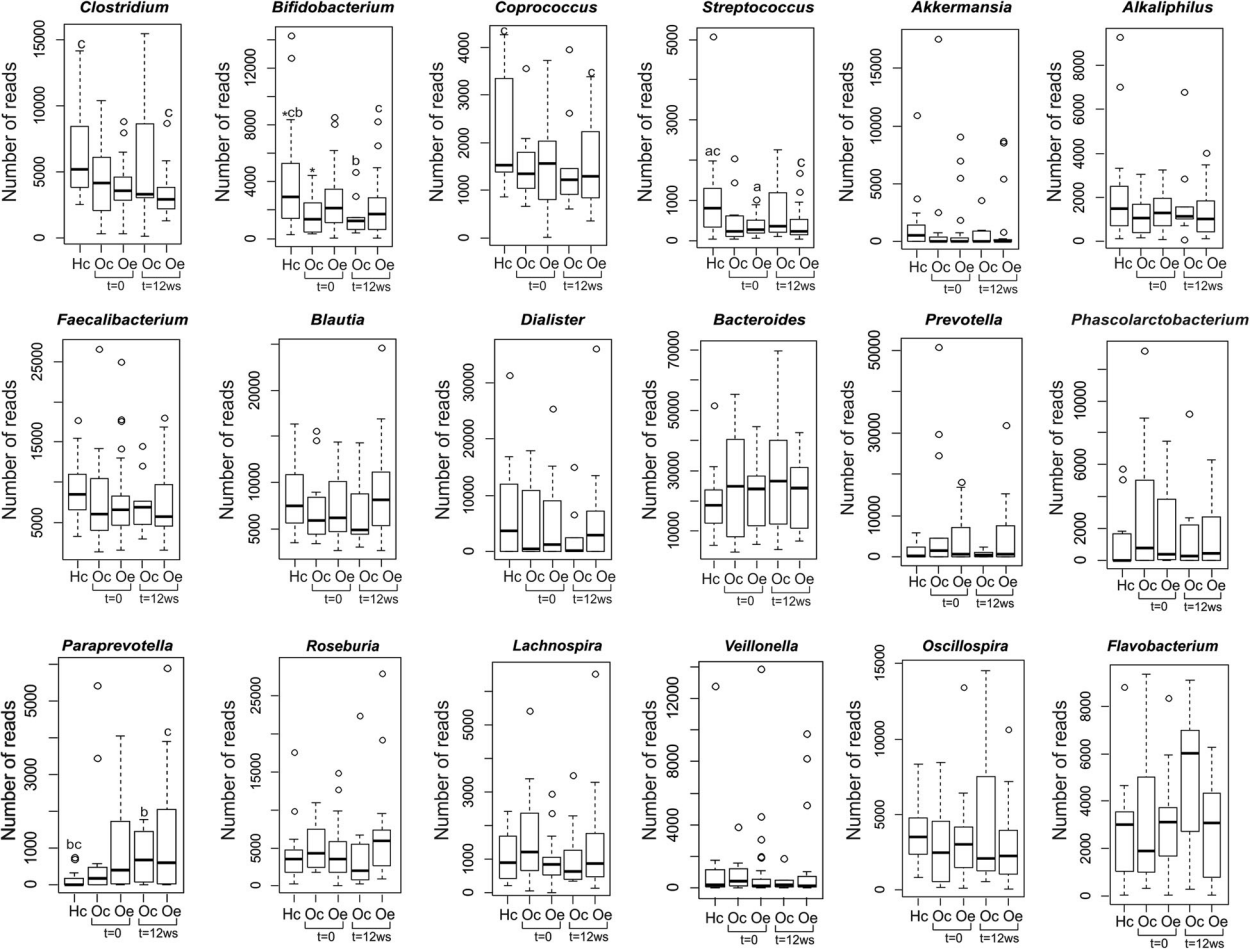
Box plots represent the differences at the genus level among heathy control children (Hc), obese control patients (Oc) and trained obese patients (Oe) at the beginning (*t* = 0) and at the end of the study (*t* = 12 weeks) using the Kruskal–Wallis test followed by the Mann–Whitney U test (*p* < 0.05). ^*^Hc vs Oc (*t* = 0), ^a^Hc vs Oe (*t* = 0), ^b^Hc vs Oc (*t* = 12 weeks), ^c^Hc vs Oe (*t* = 12 weeks).

Phylogenetic analysis of the sequences determined the presence of 549 genera of known bacteria from fecal samples from healthy control children and obese patients assigned randomly to a control or a training group. Most of the sequences were classified within six genera: *Bacteroides*, *Prevotella*, *Faecalibacterium*, *Ruminococcus*, *Blautia*, and *Clostridium*. According to the hierarchical heatmap based on the 43 genera, more representatives (Supplementary Fig. 4) and nondistinct clusters based on disease or exercise training were observed, although every individual showed a specific profile. In contrast, significant differences were observed at the genus level related to obesity and exercise training. The relative abundance of *Clostridium*, *Bifidobacterium*, *Coprococcus*, *Akkermansia*, and *Streptococcus* was significantly lower in obese patients than in healthy control children. Other genera that also showed a reduction in the relative abundance were *Alkaliphilus*, *Faecalibacterium*, *Blautia*, and *Dialister;* however, these differences were not statistically significant. However, *Bacteroides*, *Prevotella*, *Phascolarctobacterium*, and *Paraprevotella* revealed the opposite pattern (Fig. 4). It is worth mentioning that the training program tended to increase some genera, such as *Blautia*, *Dialister*, and *Roseburia*, which were reduced in obese patients, targeting a microbiota profile similar to healthy control children (Fig. 4).

### Metabolomic analysis

**Fig. 5:**
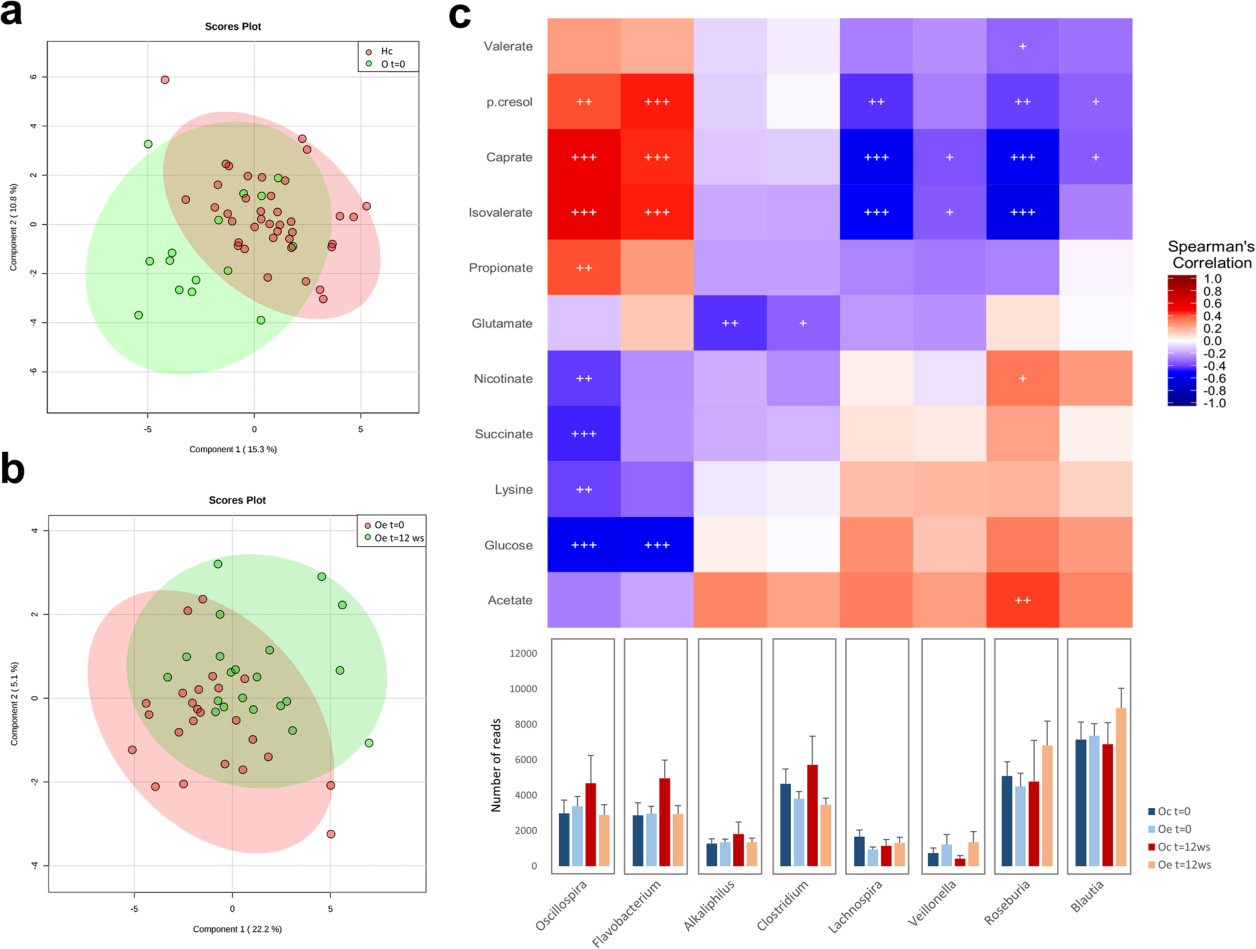
Relationship between fecal microbiota composition and metabolic profile. **a** Partial least squares-discriminant analysis (PLS-DA) of metabolites from healthy control children (Hc) and obese patients (O) at the beginning of the study. **b** PLS-DA showing the exercise performance effect on the metabolic profile in pediatric obese patients. Colored ellipses represent the 95% confidence range for the indicated experimental group. The explained variance of each component is shown in parentheses on the corresponding axis. **c** Heat map of the correlations between fecal bacterial populations at the genus and metabolite levels considering the results obtained longitudinally in all groups. Each square represents the Spearman’s correlation coefficient (*p* < 0.05). Red and blue cells specify positive and negative correlations. *p* values are corrected for multiple comparisons based on the false discovery rate (FDR).

Metabolomic analysis was performed to evaluate the metabolic differences in gut microbiota in obese control patients, trained obese patients, and healthy subjects. A total of 30 fecal metabolites were detected, including bile acids, short-chain fatty acids (SCFAs), free fatty acids, amino acids, carbohydrates, nucleotides, and organic acids. A PLS-DA method was performed to better understand the different metabolic patterns. Figure 5a shows a clear cluster formed with metabolites from all obese patients at the beginning of the study, while healthy children were dispersed. Interestingly, exercise performance modified the metabolic profile in obese patients, representing a dispersing factor (Fig. 5b) and showing a reduction in branched-chain amino acids such as isoleucine and leucine. Other metabolites, such as formate and alanine, presented a moderate reduction. Moreover, xylose, glucose, and galactose were decreased after exercise intervention (Supplementary Fig. 5).

Correlations between the differential gut microbiota composition and the fecal metabolites as an indicator of its functionality were performed, considering results obtained longitudinally in both obese groups and healthy control children. Spearman’s correlation coefficient (r) was computed for 27 different metabolites and 24 bacterial taxa (Fig. 5c). The results showed statistically significant interactions between 11 metabolites and 8 bacterial genera.

The *Lachnospira, Velionella, Roseburia*, and *Blautia* genera, which were reduced in obese patients and increased by exercise training, were shown to have a negative correlation with the fecal metabolites p-cresol, caprate, and isovalerate. *Roseburia* was also positively correlated with acetate and nicotinate (Fig. 5c). Another gut metabolome pattern was associated with some bacterial genera that were decreased by exercise training. In this case, p-cresol, caprate and isovalerate were positively correlated with the *Oscillospira* and *Flavobacterium* genera. Moreover, *Oscillospira* showed a positive correlation with propionate. In addition, both genera were negatively associated with glucose, and *Oscillospira* also showed a negative correlation with nicotinate, succinate, and lysine (Fig. 5c). Finally, *Alkaliphilus* and *Clostridium*, which were both reduced in obese patients after exercise performance, were shown to have a negative correlation with the fecal metabolite glutamate (Fig. 5c).

### Activation of inflammatory signaling pathways

**Fig. 6:**
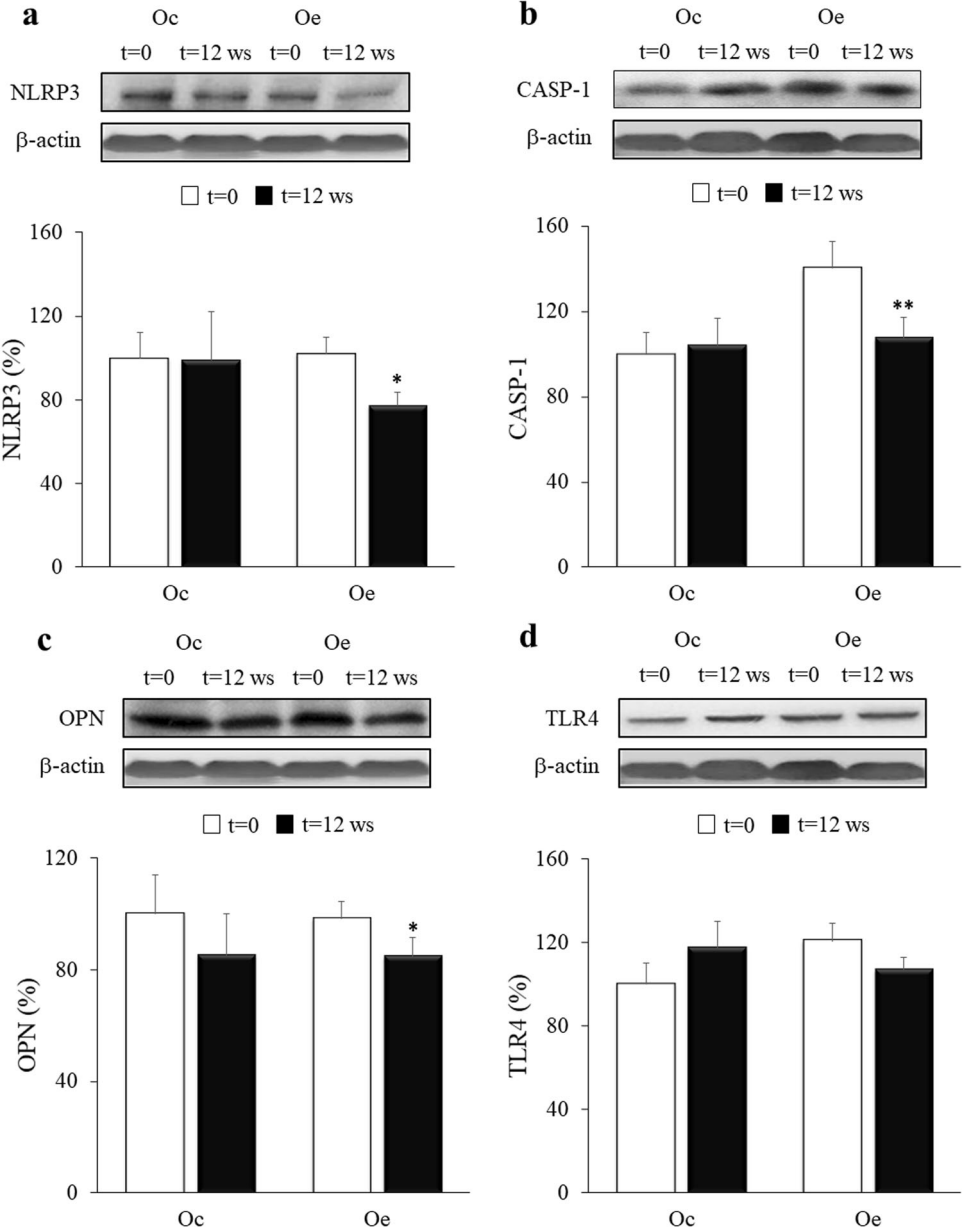
Effects of the combined strength and endurance training on NLRP3, OPN, CASP-1, and TLR4 expression. Densitometric quantification and representative western blots of NLRP3, OPN, CASP-1, and TLR4 in response to 12 weeks of combined resistance and endurance training for Oe and the same period of normal daily routines for Oc. Protein from PBMCs was separated by sodium dodecyl sulfate-polyacrylamide gel electrophoresis, followed by immunoblotting. Equal loading of proteins is illustrated by β-actin bands. Values are presented as the mean and standard error of the mean (SEM). ^*^p < 0.05; ^**^p < 0.01 vs Oe (*t* = 0).

Although the levels of caspase-1 seemed to be higher in the Oe group, no significant differences were found in the protein expression of all markers (TLR4, NLRP3, CASP-1, and OPN) at baseline between Oc and Oe (Fig. 6, Supplementary Table 1). Our results show that the signaling pathway related to inflammasome activation was significantly downregulated in the Oe group after 12 weeks of exercise training. Thus, NLRP3 protein expression was significantly reduced in the Oe group, as were the levels of active CASP-1, the last effector of the inflammasome complex. Similarly, OPN, a protein known to maintain the inflammatory state in obesity, showed a significant decrease in the group of exercised children at the end of the study. The protein levels of TLR4 revealed no significant decrease in Oe after the 12-week training program, while the opposite pattern was observed in the Oc group at the end of the experimental period. Similar results were obtained for plasma LPS levels, showing an increase in the sedentary group at the end of the study, whereas a nonsignificant reduction in the exercised group was observed (Supplementary Fig. 6). No changes in the expression of any of these proteins were found in the Oc group between both time points.

## Discussion

Because childhood obesity is one of the most serious public health concerns from this century, which is associated with metabolic syndrome and nonalcoholic fatty liver disease (NAFLD) in a mechanism that involves gut microbiota alteration, studies on the composition and functional dysbiosis of gut microbiota and on the intervention strategies have been appearing recently^28,29^. Nevertheless, there are limited and contradictory findings with regard to the composition of the gut microbiota in obese children, indicating that further analysis based on the role of the intestinal microbiota in childhood obesity is necessary^6,28^. In this regard, our results exhibited a bacterial profile associated with obesity showing a higher detection of the phylum *Proteobacteria*, while *Actinobacteria* revealed an opposite pattern compared with the healthy control group. Similar results in relation to these phyla have been found in both obese humans and mice^12,30^, confirming the role of *Proteobacteria* in obesity^30^. Previous studies have shown that the gut microbiome of obese patients is different from that of healthy-weight individuals, with a lower proportion of *Bacteroidetes* and a higher proportion of *Firmicutes*^5,29^. In our research, significant differences in the distribution of *Firmicutes* and *Bacteroidetes* were not detected between healthy and obese groups, similar to the results observed in children with NAFLD^31^. Therefore, variations between the present research and previous works, in addition to the different ages of patients, possibly reflect dissimilarity in the methodology or environmental and dietary factors, such as the degree of the disease, ethnicity or associated comorbidities and treatment. We also found significant differences among the gut bacterial populations in healthy and obese children at the genus level. Namely, decreases in the genera *Clostridium*, *Bifidobacterium*, *Coprococcus*, *Akkermansia*, and *Streptococcus* were found in obese patients in comparison with healthy children. In contrast, *Bacteroides*, *Prevotella*, *Phascolarctobacterium*, and *Paraprevotella* showed the opposite pattern. These results revealed that an aberrant gut microbiota composition might contribute to the development of childhood obesity, together with a substantial interindividual heterogeneity in the gut microbial composition, as previously proposed by other studies^32,33^.

Recent research highlights the capacity of exercise to modify the diversity, composition, and functionality of gut microbial populations^10,17,34,35^. However, there is little evidence linking exercise with gut microbial community structure modulation in humans^36^. To our knowledge, this is the first study evaluating the effects of a combined training program on metabolic status and gut microbiota composition in obese pediatric patients. Our results showed that a 12-week strength and endurance exercise intervention modified the composition and functionality of the gut microbiota, modestly affecting systemic metabolites and body composition. In this regard, several studies have shown that the receptiveness of an individual to certain lifestyle changes such as physical activity varies significantly, producing a challenge in studies designed to modify the gut microbiota^37,38^. The training program significantly decreased the relative abundance of the *Proteobacteria* phylum and *Gammaproteobacteria* class. In agreement with these findings, exercise decreased *Proteobacteria* in overweight women in response to 6 weeks of endurance training, suggesting that *Proteobacteria* is an exercise-responsive phylum^38^. In contrast, in a previous study, we reported that a combined aerobic and resistance training protocol increased the *Gammaproteobacteria* class in juvenile obese rats^10^. In addition, we observed that exercise intervention tended to increase some beneficial bacterial genera, such as *Blautia*, *Dialister*, and *Roseburia*, targeting a microbiota profile similar to healthy control children. Corroborating our findings, several studies have shown a higher detection of these genera in normal-weight subjects^39,40^. Moreover, it has been demonstrated that these genera are involved in the development of gastrointestinal and extraintestinal diseases, as well as their potential therapeutic application^40,41^. In addition, several authors have suggested that physical exercise can enhance the number of beneficial microbial species in response to homeostatic and physiological variations^42,43^. Findings from the beta-diversity estimates showed that exercise performance constitutes a dispersing factor when comparing trained obese patients with the corresponding group at the beginning of the study, which justifies the modulatory capacity of exercise on the gut microbiota composition. Similar results were shown by Allen and coworkers^34^, who reported beta-diversity variations in the microbiota in response to exercise training that were dependent on obesity status.

On the other hand, the effects of physical exercise on gut microbiota functionality were shown through the metabolic profile and represented a dispersing factor in obese patients. In this regard, changes in SCFAs, branched-chain amino acids, and several sugars were observed. Some studies have also revealed metabolomics changes during exercise performance^10,19^. Our trained patients showed a reduction in glucose levels both in plasma and in feces. Similar results have been described after a 12-week jump rope exercise program in obese adolescent girls^44^. In addition, the training protocol increased genera such as *Lachnosphira*, *Velionella*, *Roseburia*, and *Blautia*, with all of them showing a negative correlation with the fecal metabolites p-cresol and caprate. The uremic toxin p-cresol, which is increased in obese patients, has been suggested to be responsible for insulin resistance in patients on chronic dialysis^45,46^, while caprate has been described to increase the permeability of the intestinal barrier^47^. Moreover, *Roseburia*, a butyrate-producing bacterium, was also positively correlated with acetate, supporting the protective role of SCFAs in gut microbiota functionality^48^. Other genera, such as *Alkaliphilus* and *Clostridium*, were both reduced in obese children after exercise training and were negatively correlated with glutamate. This metabolite has been described as an interesting potential biomarker of abdominal obesity and metabolic risk^49^.

One of the best established interactions between the microbiota and the host is crosstalk with innate immunity^50^, mainly through Toll-like and Nod-like receptor signaling, which in turn is responsible for the low-grade inflammation state associated with obesity^14^. It is known that regular physical exercise improves the inflammatory state in children with obesity^51^, but there is no consensus on the role of TLRs in this respect. Thus, TLR4 expression has been shown to decrease in lymphocytes and monocytes after high-intensity interval training or continuous training of moderate intensity in adult women with obesity or overweight, although, as in our study, the plasma markers of inflammation remained invariable^52^. To date, there is no evidence regarding the modulation effects of exercise on gut microbiota and its association with signaling pathways of Toll and Nod type receptors in obese children. In this line, our previous study demonstrated that a high-fat diet induced a marked upregulation of TLR4 in an in vivo model of early obesity. In contrast, exercise was able to prevent LPS influx, leading to the downregulation of TLR4 after intervention in obese rats^10^. In the present research, TLR4 protein in PBMCs and plasma LPS levels showed a similar pattern in which they tended to be reduced in response to microbiota profile modulation by training, although these differences were not significant.

The reduction in the NLRP3 inflammasome and CASP-1 proteins by exercise training reinforces the idea that the NLRP3 inflammasome detects danger signals associated with obesity and contributes to obesity-induced inflammation^53^. Moreover, perturbations in the NLRP3 inflammasome could be associated with changes in gut microbiota, supporting the anti-inflammatory role of physical exercise because of its modulatory effect on gut microbiota composition and functionality in children with obesity. These data are in agreement with another work that showed how NLRP3 inflammasome activation triggered by intestinal dysbiosis in mice was restored to control levels by quercetin supplementation, a flavonoid with anti-inflammatory properties^11^.

On the other hand, OPN acts as an inflammatory cytokine through a variety of different receptors in certain inflammatory disorders such as obesity^54^. In fact, OPN seems to play an important role in the development of adipose tissue inflammation and insulin resistance, although the mechanisms by which it performs this function remain to be clarified^54^. In our study, 12 weeks of combined strength and endurance training reduced PBMC OPN protein levels in obese children. Similarly, previous studies showed that serum OPN significantly decreased in obese young women who participated in an exercise program for 8 weeks. However, these reduced OPN contents did not correlate with body fat percentage, as occurred in our work, suggesting that OPN may be controlled by other different factors in obese humans^55^. Moreover, it has been suggested that this protein could be involved in gut microbiota regulation^56^.

It is widely demonstrated that regular physical activity is healthy and induces significant improvements in quality of life. In the case of obese children, most studies suggest that physical exercise is a key tool for reducing abdominal obesity or cardiovascular risk and improving metabolic parameters^16^. In our research, after 12 weeks of the combined strength and endurance training program, one of the beneficial effects of exercise was a significant increase in strength in the upper and lower extremities in the obese trained group. After the intervention, the maximum dynamic strength percentage change was two times and four times higher in the lower and upper extremities, respectively, in the experimental group because of exercise training. These changes show the effectiveness at the functional level of the strength training that was carried out. The greatest difference in the increase in strength in the upper extremities is explained by the fact that the lower extremities are commonly used in daily life (body weight with or without class backpacks), while often the upper limbs are not exercised with sufficient overload beyond the weight of one’s extremities. These data corroborate previous results obtained by other authors comparing obese children with sedentary and active lifestyles^57^. Moreover, several studies have demonstrated a possible role for gut microbiota in the maintenance of muscle strength, as well as in the adaptation of physiological functions and exercise capacity^58,59^. These findings could justify the possible connection between our changes in gut microbiota composition and functionality and the beneficial effects of exercise. However, our results did not reveal significant differences in the anthropometric parameters analyzed in the obese group after 12 weeks because of the exercise intervention. Several reasons could explain the absence of a modulatory effect of the training protocol on body weight and composition. This study was carried out in children and, therefore, in the growth phase. Although all participating children received healthy eating and lifestyle guidelines, it is possible that they did not follow the recommendations and advice properly. Moreover, it was not expected that physical exercise would produce loss of body weight or fat mass *per se* because most of the studies that show significant weight loss in a population of children have used longer training programs than ours^60^.

This study presents a number of limitations. First, the absence of normal-weight children restricts the interpretation of the effects of combined training on the inflammatory response. The authors decided not to include this group given the difficulty of finding healthy children for the extraction of blood samples. On the other hand, pediatric patients usually suffer pathological processes that can strongly condition the inflammatory process and, therefore, alter the results. Second, it would be necessary to increase the sample size to obtain more statistical power, especially in those parameters that show a tendency to change. In addition, the link between changes in the gut microbiota composition and the modulation of inflammatory pathways induced by exercise has not been completely demonstrated, and more experiments are necessary to support this relationship. Finally, it is important to emphasize that participants were not subjected to a caloric restriction diet but received nutritional advice for a healthy and balanced diet. This fact could also explain the lack of significant differences between the control and trained obese groups, mainly in the anthropometric and biochemical variables.

In conclusion, our findings suggest the existence of a deleterious microbiota profile in obesity that is positively modified by exercise intervention, highlighting the value of exercise performance as an efficient nonpharmacological therapy in early obesity. Nevertheless, further studies are needed to fully understand the mechanisms that determine the changes in the composition and functionality of the microbiota caused by exercise and all their related effects.

## Supplementary information

Supplementary Information
