# Supplementary material for: Exercise training modulates the gut microbiota profile and impairs inflammatory signaling pathways in obese children
Source: Exp Mol Med. 2020 Jul 6;52(7):1048–61. doi: 10.1038/s12276-020-0459-0 (PMC8080668; doi:10.1038/s12276-020-0459-0)
Supplement: Supplementary file 1 — Supplementary Information [file 12276_2020_459_MOESM1_ESM.docx]

**Supplementary information**

**EXERCISE TRAINING MODULATES THE GUT MICROBIOTA**

**PROFILE AND IMPAIRS INFLAMMATORY SIGNALING**

**PATHWAYS IN OBESE CHILDREN**

Rocío Quiroga^1^, Esther Nistal^1,2^, Brisamar Estébanez^2^, David Porras^2^, María Juárez-Fernández^2^, Susana Martínez-Flórez^2^, María Victoria García-Mediavilla^2,3^, José A. de Paz^2^, Javier González-Gallego^2,3^, Sonia Sánchez-Campos^2,3*^, María J. Cuevas^2*^

1. Complejo Asistencial Universitario (CAULE), León, Spain

2. Institute of Biomedicine (IBIOMED), León, Spain

3. Centro de Investigación Biomédica en Red de Enfermedades Hepáticas y Digestivas (CIBERehd), Madrid, Spain

* These authors share senior authorship

**SUPPLEMENTARY INFORMATION**

[**Supplementary Table 1** 2](#_Toc39591496)

[**Supplementary Figure 1** 3](#_Toc39591497)

[**Supplementary Figure 2** 4](#_Toc39591498)

[**Supplementary Figure 3** 5](#_Toc39591499)

[**Supplementary Figure 4** 6](#_Toc39591500)

[**Supplementary Figure 5** 7](#_Toc39591501)

[**Supplementary Figure 6** 8](#_Toc39591502)

# Supplementary Table 1 Quantitative results of the Western blot densitometry quantification of NLRP3, CASP-1, OPN and TLR4 proteins before (t=0) and after (t=12 ws) of the 12-week period of combined strength and resistance training in control (Oc) and trained (Oe) obese children.

|  | **Oc t=0**  **Mean ± SEM** | **Oe t=0**  **Mean ± SEM** | ***p* value t=0**  **Oc *vs* Oe** | **Oc t=12 ws**  **Mean ± SEM** | ***p* value Oc t=12 ws *vs* t=0** | **Oe t=12 ws**  **Mean ± SEM** | ***p* value Oe**  **t=12 ws *vs* t=0** |
| --- | --- | --- | --- | --- | --- | --- | --- |
| **NLRP3** | 100.000 ± 11.994 | 102.078 ± 7.933 | 0,168 | 99.021 ± 14.736 | 0.945 | 77.344 ± 9.747 | 0.014^*^ |
| **CASP-1** | 100.000 ± 16.768 | 140.109 ± 11.091 | 0,887 | 103.883 ± 14.597 | 0.797 | 107.231 ± 9.655 | 0.003^**^ |
| **OPN** | 100.000 ± 10.717 | 98.401 ± 7.088 | 0,059 | 84.994 ± 11.699 | 0.139 | 84.874 ± 7.738 | 0.048^*^ |
| **TLR4** | 100.000 ± 2.450 | 103.981 ± 1.619 | 0,902 | 103.478 ± 1.969 | 0.242 | 101.640 ± 1.290 | 0.235 |

Values are represented as mean and standard error of the mean (SEM). ^*^*p* < 0.05; ^**^*p* < 0.01

# Supplementary Figure 1


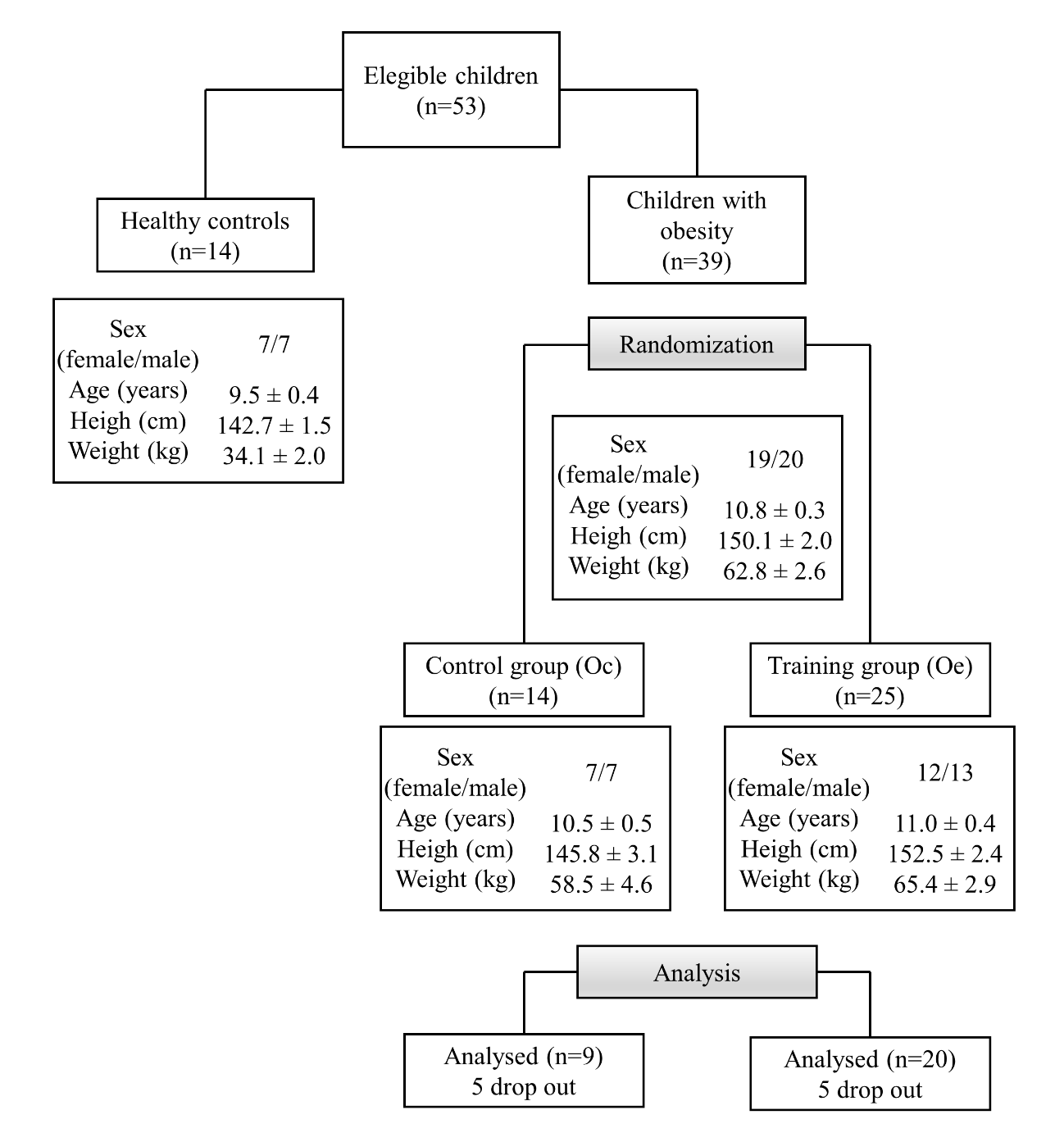


**Supplementary Figure 1** Characteristics of patients.

# Supplementary Figure 2


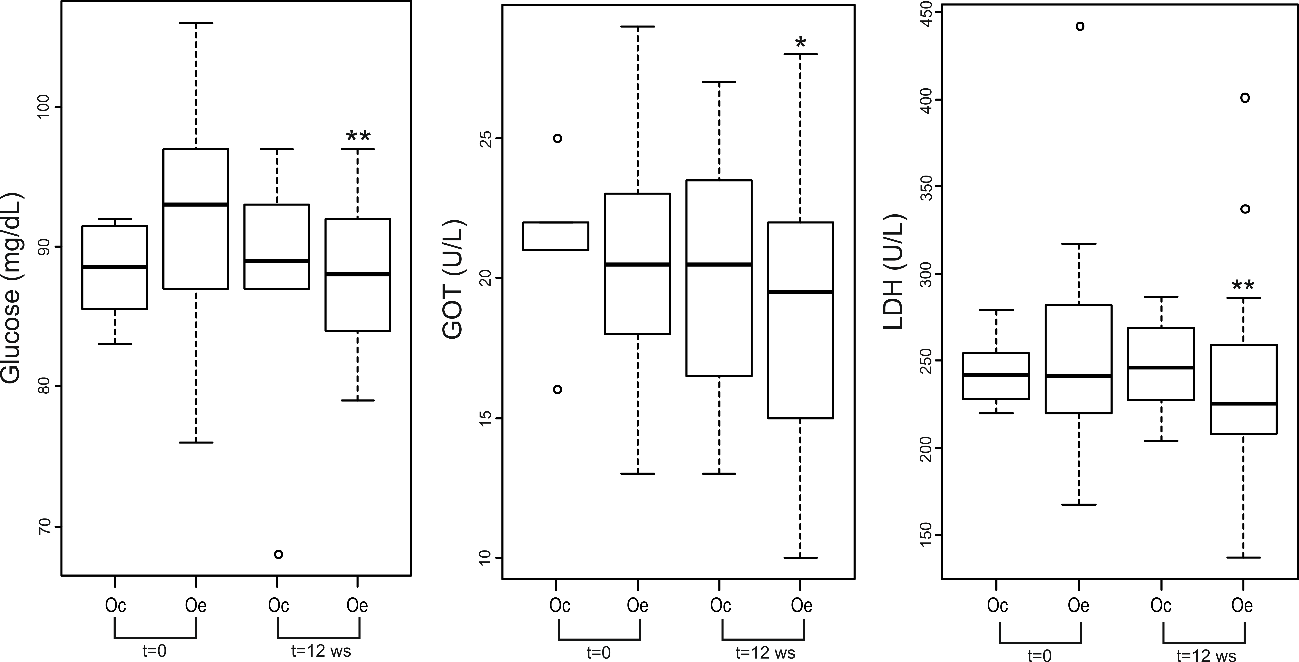


**Supplementary Figure 2** Box plots represent the differences among obese control patients (Oc) and training obese patients (Oe) at the beginning (t=0) and the end of the study (t=12 ws) in glucose (mg/dL), lactate deshydrogenase (LDH) (U/L) and glutamate-oxaloacetate transaminase (GOT) (U/L) plasma levels, using Wilcoxon test; ^*^*p*<0.05 *vs* Oe (t=0); ^**^*p*<0.01 *vs* Oe (t=0).

# Supplementary Figure 3


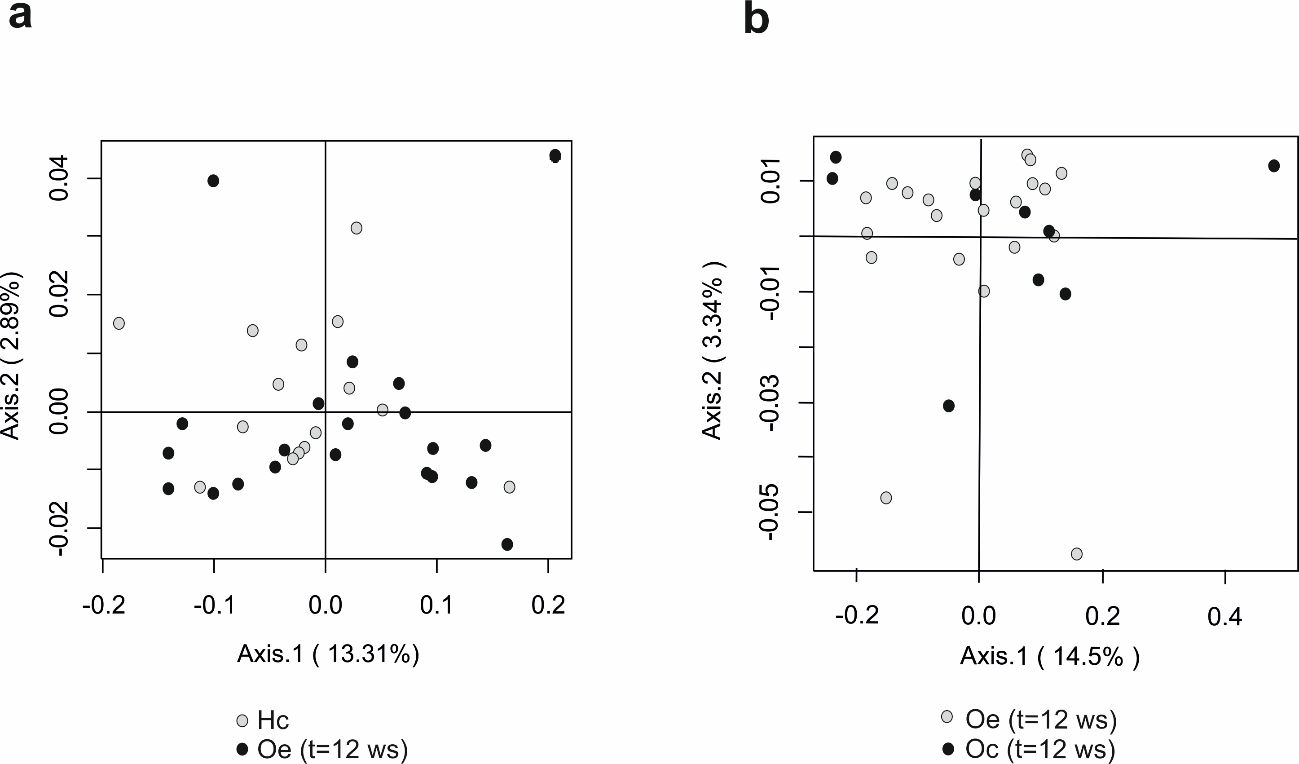


**Supplementary Figure 3** Principal Coordinates Analysis (PCoA) plot derived from the Morisita-Horn index at the phylum level **a)** between healthy control children (Hc) and obese training patients (Oe, t=12ws) and **b)** between obese control patients (Oc, t=12ws) and obese training patients (Oe, t=12ws). The percentage of the total variance explained is indicated in parenthesis in each axis.

# Supplementary Figure 4


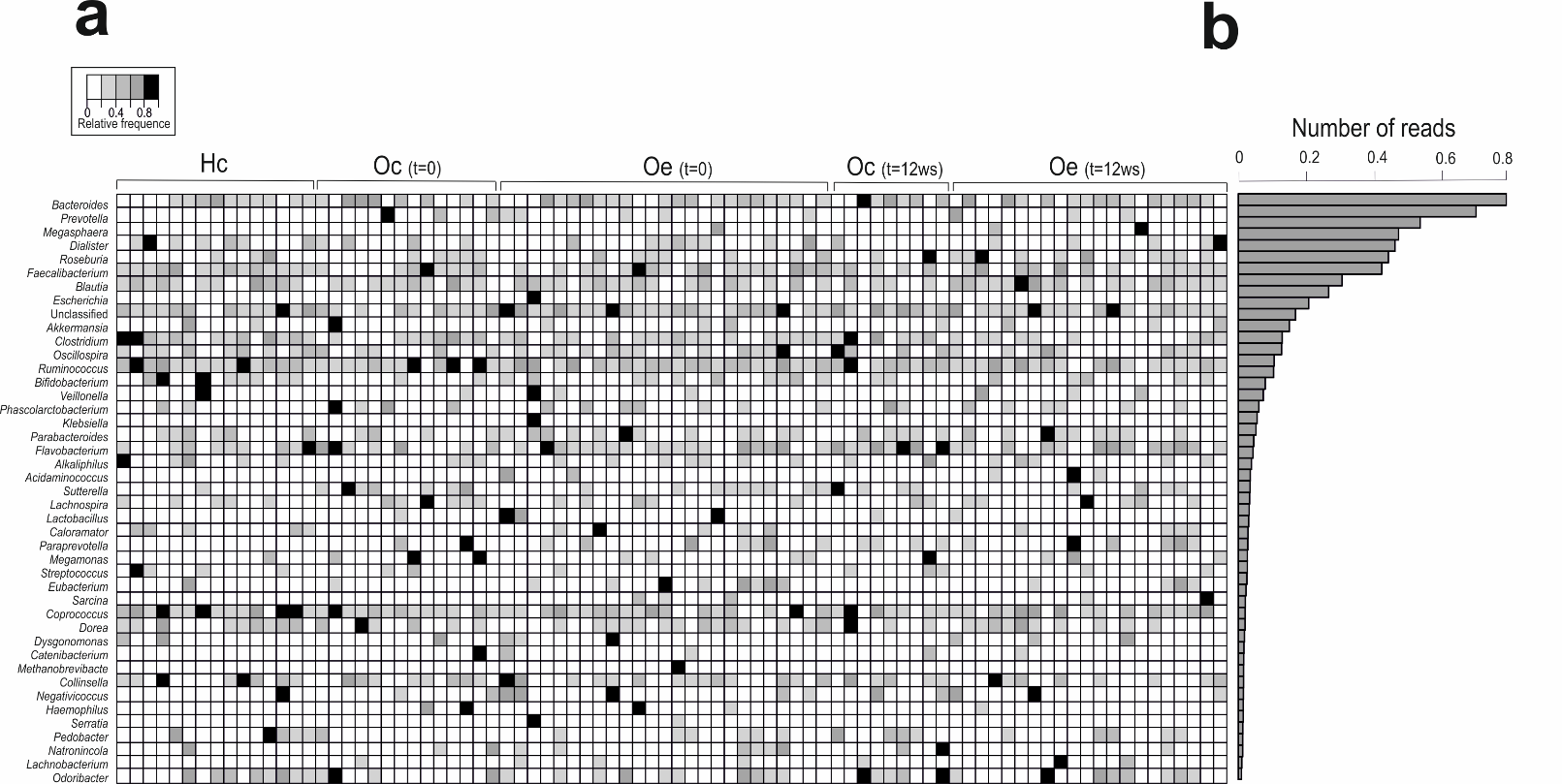


**Supplementary Figure 4** Microbiota heatmap at genus level. **a)** The heatmap plot represents the log of the relative frequency of each OTU (right vertical axis) within each faecal sample (horizontal axis). The gray level scale intensity according to the legend in the top left corner specifies the log of the relative values for each OTU. The assignment of each OTU was performed using the Naïve Bayesian classifier provided by the Ribosomal Database Project (RDP). **b)** Histogram representing number of reads.

**Supplementary Figure** 5

**a**


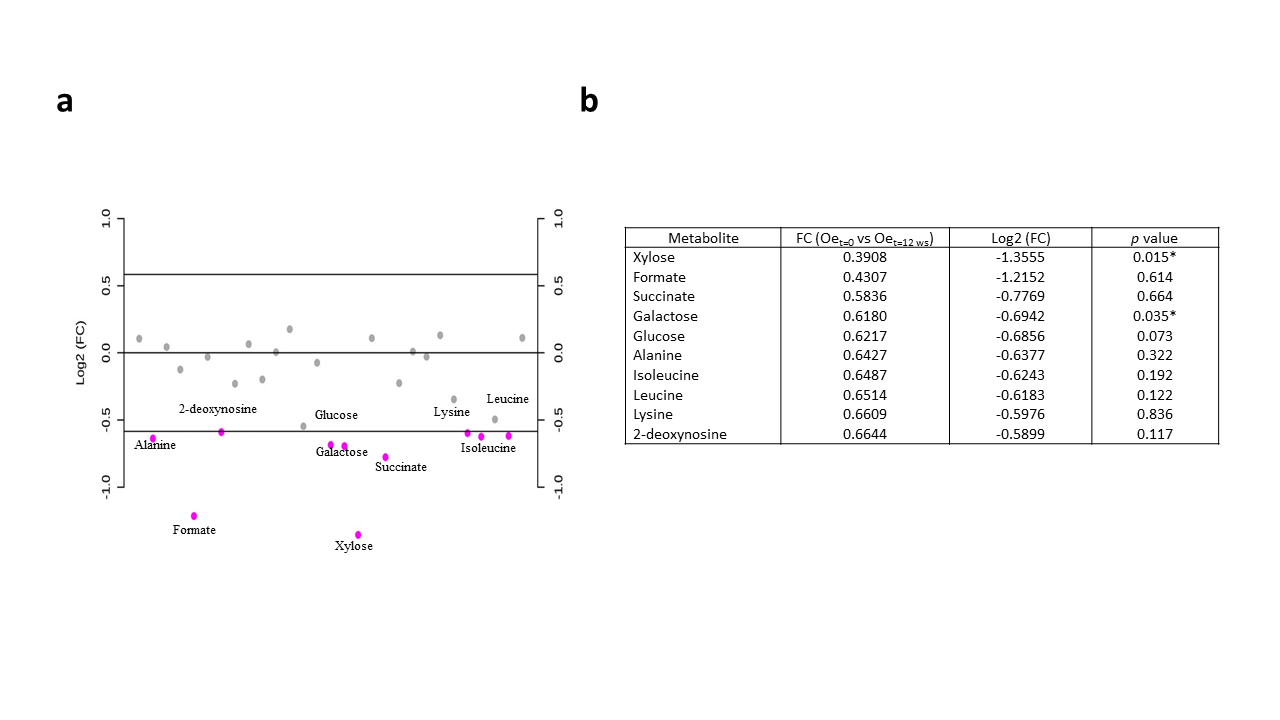

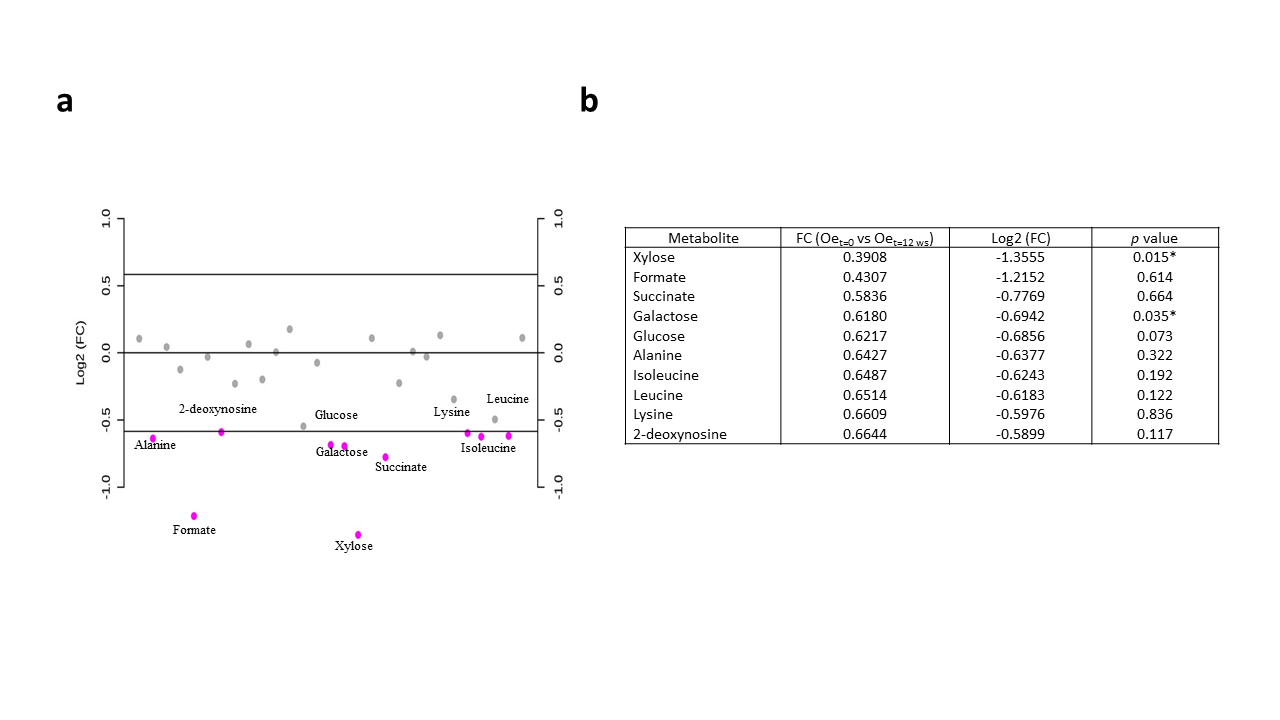


**b**

**Supplementary Figure 5 a)** Plot of fold change (FC) analysis of metabolites between obese children before (Oe, t=0) and after the completion of the physical training protocol (Oe, t=12 ws). **b)** Table summarizes metabolites with FC > 1,5. Wilcoxon rank sum test was employed to determine significant changes: **p*<0.05.

# **Supplementary Figure 6**

**
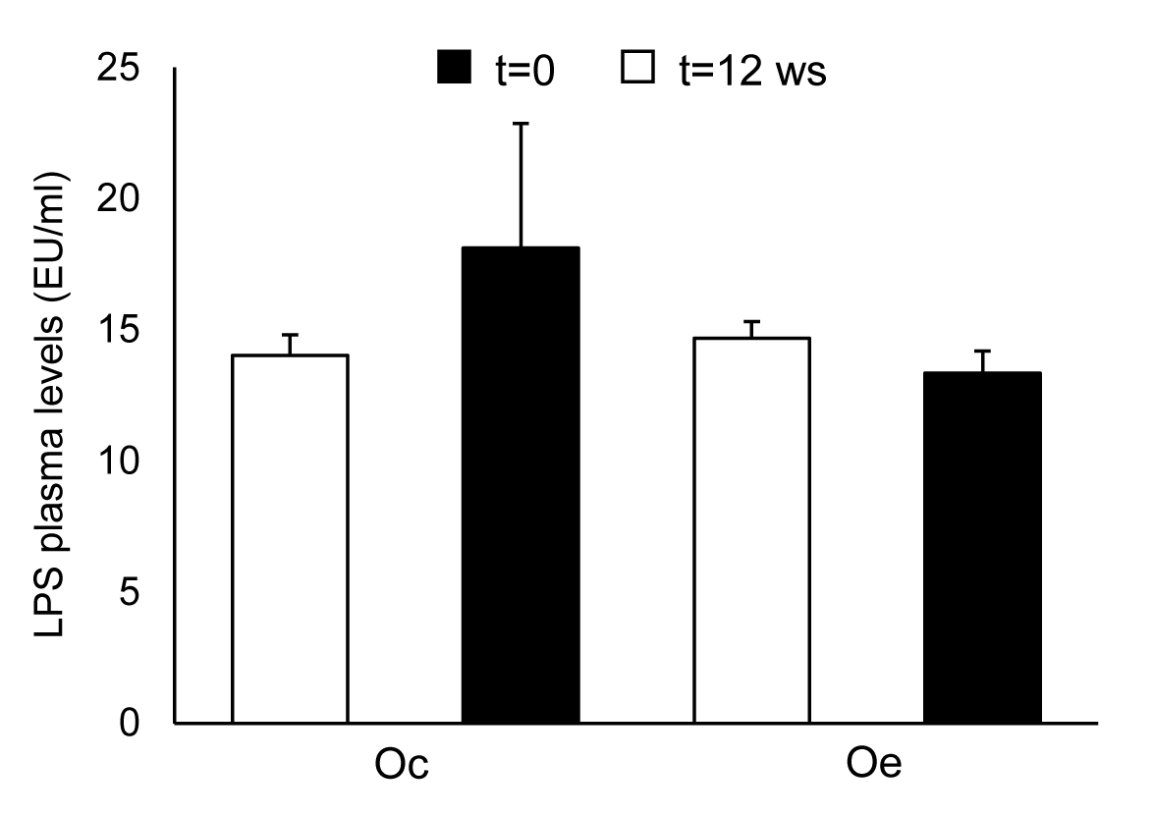
**

**Supplementary Figure 6** Determination of LPS levels in plasma samples of obese sedentary (Oc) and obese trained (Oe) groups at t=0 and at the endpoint of the study (t=12ws) by the LAL chromogenic assay.
